# Assembly strategies for rubber-degrading microbial consortia based on omics tools

**DOI:** 10.3389/fbioe.2023.1326395

**Published:** 2023-12-06

**Authors:** Chengda Cui, Mengke Jiang, Chengxiao Zhang, Naxue Zhang, Feng-Jie Jin, Taihua Li, Hyung-Gwan Lee, Long Jin

**Affiliations:** ^1^ Co-Innovation Centre for Sustainable Forestry in Southern China, College of Ecology and Environment, Nanjing Forestry University, Nanjing, China; ^2^ Cell Factory Research Centre, Korea Research Institute of Bioscience and Biotechnology (KRIBB), Daejeon, Republic of Korea

**Keywords:** natural rubber, polyisoprene, rubber-degrading bacteria, microbial consortia, metagenomics, omics

## Abstract

Numerous microorganisms, including bacteria and fungus, have been identified as capable of degrading rubber. Rubber biodegradation is still understudied due to its high stability and the lack of well-defined pathways and efficient enzymes involved in microorganism metabolism. However, rubber products manufacture and usage cause substantial environmental issues, and present physical-chemical methods involve dangerous chemical solvents, massive energy, and trash with health hazards. Eco-friendly solutions are required in this context, and biotechnological rubber treatment offers considerable promise. The structural and functional enzymes involved in poly (cis-1,4-isoprene) rubber and their cleavage mechanisms have been extensively studied. Similarly, novel bacterial strains capable of degrading polymers have been investigated. In contrast, relatively few studies have been conducted to establish natural rubber (NR) degrading bacterial consortia based on metagenomics, considering process optimization, cost effective approaches and larger scale experiments seeking practical and realistic applications. In light of the obstacles encountered during the constructing NR-degrading consortia, this study proposes the utilization of multi-omics tools to discern the underlying mechanisms and metabolites of rubber degradation, as well as associated enzymes and effective synthesized microbial consortia. In addition, the utilization of omics tool-based methods is suggested as a primary research direction for the development of synthesized microbial consortia in the future.

## 1 Introduction

Polyisoprene is classified as one of the eight principal categories of biopolymers that are synthesized by living microbes. NR, one of the representatives of the polyisoprene group, is widely employed among biopolymers. By coagulating and curing the latex of rubber plants, NR is transformed into an elastomer (Steinbüchel, 2001; Linos and Steinbüchel, 2005; Lynen et al., 1958). Presently, over 2,500 plant species are known to produce NR. On the contrary, the rubber tree *Hevea brasiliensis*, which is exclusive to subtropical regions, is the most significant commercial source of this polymer (Nair, 2010), supplying 99% of the global market. Melaleuca (*Parthenium argentatum*) and Russian dandelion (*Taraxacum kok-saghyz*) are two additional sources. Asia currently accounts for 92% of NR production, with Thailand leading the world, followed by Indonesia, Malaysia, India, Vietnam, and China.

**FIGURE 1:**
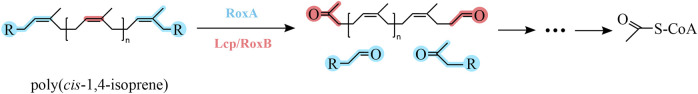
Poly (cis-1,4-isoprene) metabolic pathway of microbial degradation. Rubber oxigenases (Lcp/RoxA/RoxB) are responsible for the first cleavage step of poly (cis-1,4-isoprene) chains. The chemical structure was illustrated with ChemDraw 22.

NR is a 2-methyl-1, 3-butadiene polymer. It is a conjugated diene with double bonds in alternating locations, often known as polyisoprene. Cis-1,4-polyisoprene accounts for 90% of the dry weight of NR. Source-dependent variations exist in the molecular weight and composition of this polymer (Mooibroek and Cornish, 2000). Cis-1,4-polyisoprene rubber is a crucial biological material in contemporary society. In the context of commercial applications, this polymer typically experiences vulcanization, which modifies its molecular structure via the crosslinking of isoprene chains (Figure 1). Tires are vulcanized through the application of heat in the presence of sulfur, while latex mittens are vulcanized via peroxidation and irradiation. Rubber commonly used to construct wire coverings, mittens, and footwear is produced via this method. Moreover, in order to impart rigidity, elasticity, and longevity to unprocessed latex, a multitude of chemical constituents are incorporated into it. As a result, it finds application in industries such as manufacturing, transportation, medicine, and domestic products.

The physical and chemical properties of NR are highly stable and difficult to undergo natural degradation, thus leading to the accumulation of large amounts of NR waste and pending disposal. Consequently, the environmental contamination of byproducts resulting from the extensive utilization of NR has emerged as a significant global concern. At this stage, incineration and landfill methods are mainly used to dispose of NR waste, and this traditional treatment method has undoubtedly caused serious pollution to the environment (Mooibroek and Cornish, 2000). It presents a significant environmental contamination problem, poses a critical risk to human health by triggering allergies and asthma, and the incineration process generates substantial quantities of atmospherically hazardous toxic gases (e.g., sulfur oxides, carbon monoxides, and cyanides) (Depaolini et al., 2017; Kasai, 2020; Chittella et al., 2021). As environmental contamination has increased, the utilization of microorganisms to degrade contaminants has emerged as a major concern. In recent years, researchers have explored the ability of some microorganisms to degrade NR in laboratory studies and practical applications and to break down rubber waste into easily degradable and environmentally benign substances.

This review focused on constructing NR degrading functional microbial consortia. We briefly reviewed its properties and biodegradation pathways, as well as present research gaps and recent achievements in NR biodegradation. Then, in comparison to single strains, we examined the benefits of employing microbial functional consortia and their potential for NR degradation. Finally, we looked at the possibility of applying multi-omics tool to develop functional microbial consortia.

## 2 Rubber biodegradation by single microorganisms

### 2.1 Microbial degradation mechanisms of rubber

The earliest report on the degradation of rubber by microbes was published in 1914, when Söhngen and Fol utilized highly pure NR as the only carbon source to demonstrate the absorption of the hydrocarbon chain by microorganisms (Söhngen and Fol, 1914). Two *Actinomycetes* were discovered to form colonies on the purified rubber material. Spence and Van Niel (1936) first isolated bacteria capable of degrading rubber by employing latex overlay plates. The formation of translucent halos within the opaque agar layer indicated the beginning of colony formation. The clear zone isolation technique enables the detection of bacteria that secrete extracellular enzymes during the degradation of cis-1,4-polyisoprene. This method selects NR utilizing strains based on the formation of translucent halos (hyaline zones) around colonies on opaque NR agar plates, which can be directly observed by the naked eye after the degradation action of the strain. Members of these groups usually belong to the class of *Actinomycetes* that can form mycelium, such as *Actinobacteria*, *Streptomyces*, and *Micromonospora*. In contrast, the second group of microorganisms that degrade NR do not produce any clear zones, and only the normal growth of strains can be observed on NR agar plates without forming any clear circles around the colonies. Therefore, these strains need to be in direct contact with the rubber matrix to function. These bacteria attack the rubber matrix directly, form biofilms and bind to polymers, which begin to degrade at the cell surface. Examples of this group are *Nocardia*, *Mycobacterium*, and *Gordonia* (Gallert, 2000).

### 2.2 Microorganisms involved in rubber degradation (bacteria and fungi)

At present, researchers have found that some microorganisms with special origins have some biodegradation effect on NR, and the microbial degradation of NR has been studied by extracting strains from specific environments (Tables 1, 2). *Actinomycete* isolated from sludge at wastewater treatment plants could grow and multiply in liquid medium using NR as a carbon source, and it was confirmed that the isolated strains could reduce the molecular weight of NR thus achieving microbial degradation of NR; *Gordonia* and *Actinomycete* (Hiessl et al., 2012; Ali Shah et al., 2013) isolated from soil at rubber plantations were found to be effective in microbial degradation of NR; the use of insect laccase and manganese peroxidase secreted by *Penicillium* can also be used for the biodegradation of NR (Nayanashree and Thippeswamy, 2015b). It has been reported that *Rhodococcus*, the predominant species accountable for the degradation of NR, is capable of degrading organic pollutants and is therefore considered effective (Dey et al., 2020). *Streptomyces* have been studied by researchers to degrade non-vulcanized fresh latex and common vulcanized rubber products such as NR gloves, NR condoms and rubber car tires (Nanthini and Sudesh, 2017). *Variovorax* degrades ethylene glycol phthalate (Prasad, 2017) that can be used as a plasticizer for plastic and rubber products and is used as a component of NR products with improved strength. It is presumed that strains of this genus can also degrade NR. *Pseudomonas* has been shown to make use of NR hydrocarbons and subsequently can degrade NR (Roy et al., 2006).

**TABLE 1:** Reported bacterial strains with a rubber degradation capacity.

Genus	Isolation source	Culture time	Weight loss	Rubber type	References
*Achromobacter*	Soil sample from Aswan, Egypt	60 days	5.9%	Tire rubber	Berekaa et al. (2005)
*Acinetobacter*	sewage	10 weeks	12%	Vulcanized rubber	Bode et al. (2001)
*Actinomadura*	Food residues of animal husbandry in Egypt	60 days	7%	Natural rubber latex	Ibrahim et al. (2006), Vivod et al. (2019), Sarkar et al. (2022)
*Actinoplanes*	Soil	30 days	–	Natural rubber	Imai et al. (2011)
*Amycolatopsis*	Soil sample	6 weeks	0.1%	Vulcanized rubber	Heisey and Papadatos (1995), Chengalroyen and Dabbs (2013)
*Bacillus*	Latex contaminated soil	4 weeks	–	Natural latex rubber	Cherian and Jayachandran (2009)
*Gordonia*	Sewage from deteriorated car tires	5 weeks	–	Natural and synthetic rubber	Linos et al. (2000)
*Methylibium*	Soil samples from Japan	70 days	–	Latex dispersed in agar	Imai et al. (2011)
*Micromonospora*	Sewage from deteriorated car tires	5 weeks	–	Natural and synthetic rubber	Berekaa et al. (2000), Linos et al. (2000)
*Mycobacterium*	Sewage from deteriorated car tires	5 weeks	–	Natural and synthetic rubber	Berekaa et al. (2000), Linos et al. (2000)
*Nocardia*	Sandy soil and industrial waste	6–8 weeks	10%–55.3%	Natural and synthetic rubber	Ibrahim et al. (2006), Vivod et al. (2019), Sarkar et al. (2022)
*Nocardioides*	Soil	–	–	Poly(cis-1,4-isoprene)	Gibson et al. (2020)
*Nonomuraea*	Soil	4 weeks	–	Natural rubber	Basik et al. (2021a)
*Paenibacillus*	Soil	8 weeks	–	Natural rubber	Hapuarachchi et al. (2016)
*Pseudomonas*	Soil sample from the ground of waste tires	2 months	43.1%/10.8%	Natural and vulcanized rubber	Roy et al. (2006), Bosco et al. (2018)
*Rhizobacter*	Soil sample in a botanical garden in Japan	70 days	–	Poly(cis-1,4-isoprene)	Imai et al. (2011), Imai et al. (2013)
*Rhodococcus*	Rubber processing plant wastewater	–	–	Vulcanized rubber (glove)	Watcharakul et al. (2016)
*Steroidobacter*	DSM 103114	7 days	60%	Natural rubber	Tsuchii and Takeda (1990), Sharma et al. (2018)
*Streptomyces*	Soil	30 days	–	Natural rubber	Hesham et al. (2015)
*Xanthomonas*	Soil	10 weeks	12%	Natural latex, synthetic rubber	Bode et al. (2001), Jendrossek and Reinhardt (2003)

**TABLE 2:** Reported fungal isolates with a rubber degradation capacity.

Taxa	Isolation source	Culture time	Weight loss	Rubber type	References
*Alternaria alternata*	Soil suspension	160 days	–	Natural rubber	Bosco et al. (2018)
*Aspergillus niger*	Latex sample and leafs	30 days	28.0%	Natural rubber	Mohamed et al. (2017)
*Ceriporiopsis subvermispora*	*Quercus* sp. log	200 days	2.70%	Natural rubber	Sato et al. (2004)
*Cladosporium cladosporioides*	Soils and deteriorated tyres and rubber	6 months	–	Natural rubber	Borel et al. (1982)
*Fusarium solani*	Soil	10 days	–	Natural rubber	Borel et al. (1982)
*Monascus rubber*	Crude rubber specimen of Hevea brasiliens	2 weeks	–	Crude rubber of Hevea brasiliensi	Schade (1937)
*Monascus purpureus*	Crude rubber specimen of Hevea brasiliens	2 weeks	–	Crude rubber of Hevea brasiliensi	Schade (1937)
*Mucor*	Effluents and natural rubber waste serum	8 days	–	–	Atagana* et al. (1999)
*Paecilomyces lilacinus*	Soils and deteriorated tyres and rubber	20 days	–	Natural rubber	Borel et al. (1982)
*Penicillium chrysogenum*	Latex sample and leafs	30 days	40%	Natural rubber	Mohamed et al. (2017)
*Penicillium variable*	Permafrost deposits	70 days	15%	polyisoprene	Joseph et al. (2022)
*Phoma eupyrena*	Soils and deteriorated tyres and rubber	20 days	–	Natural rubber	Borel et al. (1982)
*Phlebia radiata*	Wood	–	–	–	Singh (2017)
*Resinicium bicolor*	Conifer	8 days	–	Cryo-ground tire rubber	Bredberg et al. (2002)
*Rhodotorula mucilaginosa*	Soil suspension	160 days	–	Natural rubber	Bosco et al. (2018)

### 2.3 Rubber degradation enzymes

The two major NR degrading enzymes rubber oxygenase (RoxA/RoxB) and latex clearing protein (Lcp) have been identified in Gram-negative and Gram-positive bacteria, respectively (Table 3). Among Gram-positive NR degrading bacteria, Lcp is responsible for the initial oxidative cleavage of cis-1,4-polyisoprene in the strains, first demonstrated in *Streptomyces* sp. K30. The Lcp of this strain was experimentally shown to be a type B cytochrome that cleaves cis-1,4-polyisoprene to a low molecular weight product containing isoprene with aldehyde and ketone termini. The resulting compound is further degraded to the corresponding acid by oxidoreductase OxiAB, which is encoded by the OxiAB gene and subsequently enters the β-oxidation pathway via the action of coenzyme A for eventual oxidative catabolism. Yikmis et al. found that transcription of Lcp increases in the presence of latex or synthetic cis-1,4-polyisoprene and that Lcp transported via the bisarginine transport (Tat) system is secreted (Jendrossek and Reinhardt, 2003; Yikmis et al., 2008; Imai et al., 2011; Imai et al., 2013; Sharma et al., 2018), thus confirming the role of Lcp in the degradation of NR. In Gram-negative NR degrading bacteria, including some *mycobacteria*, the rubber oxygenase RoxA is responsible for the degradation of cis-1,4-polyisoprene. RoxA, encoded by the *roxA* gene, is an exo-type and a highly representative rubber oxygenase that occupies the same important position as the Lcp. It was first isolated and confirmed in strain *Xanthomonas* sp. 35Y. RoxA was shown to be a key enzyme involved in the degradation of cis-1,4-polyisoprene in strain *Xanthomonas* sp. 35Y. RoxA first binds oxygen molecules and then cleaves the carbon-carbon double bond of cis-1,4-polyisoprene to produce isoprene with aldehyde and ketone groups at the end of the molecule (Braaz et al., 2004; Hiessl et al., 2014; Luo et al., 2014). RoxA is an extracellular dioxygenase that oxidize cis-1,4-polyisoprene to 12-oxo-4,8-dimethyltridecane-4,8-diene-1-aldehyde (ODTD) thereby degrading NR. Conversely, the rubber oxygenase RoxB is also involved in the degradation process, which degrades NR by endo-cleavage of cis-1,4-polyisoprene. It is known from the experimental results of Daisuke Kasai et al. that the LatA protein is a key enzyme for the degradation of NR in strain NBRC 109400 demonstrated by the high-throughput transposon insertion technique (Birke et al., 2013; Seidel et al., 2013; Kasai et al., 2017). The direct relatives of functional RoxB and RoxA, namely, LatA1 and LatA2, respectively, were reported to be essential for NR degradation in *Rhizobacter gummiphilus* NS21 (Linos et al., 2000; Ibrahim et al., 2006; Yikmis and Steinbüchel, 2012).

**TABLE 3:** Characteristics of NR degrading enzymes.

Enzyme	RoxA	RoxB	Lcp
Identified from	*Algiphilus aromaticivorans* DG1253	*Alcanivorax* sp. MD8A	*Actinoplanes* sp. OR16
	*Archangium* sp. Cb G35	*Alcanivorax* sp. NORP25	*Dactylosporangium* sp. AC04546
	*Archangium violaceum* Cb vi76	*Alcanivorax* sp. P2S70	*Gordonia polyisoprenivorans* VH2
	*Burkholderia* sp. Bp7605 MSMB0175	*Alcanivorax hongdengensis* A-11-3	*Gordonia westfalica* Kb1
	*Burkholderia* sp. MSMB175	*Alcanivorax nanhaiticus* 19-m-6	*Microtetraspora* sp. AC03309
	*Burkholderia singularis* LMG 28154	*Archangium* sp. Cb G35	*Nocardia farcinica* E1
	*Chondromyces apiculatus* DSM 436	*Archangium violaceum* Cb vi76	*Nocardia farcinica* NVL3
	*Corallococcus coralloides* SK5025	*Burkholderia ubonensis* MSMB1191	*Rhodococcus rhodochrous* RPK1
	*Corallococcus coralloides* DSM 2259	*Burkholderia ubonensis* MSMB1137WGS	*Streptomyces* sp. AC04842
	*Haliangium ochraceum* DSM 14365	*Corallococcus coralloides* DSM 2259	*Streptomyces* sp.K30
	*Microbulbifer mangrovi* DD-13	*Fontimonas thermophila* DSM 23609	
	*Minicystis rosea* DSM 24000	*Haliangium ochraceum* DSM 14365	
	*Myxococcus fulvus* 124B02	*Hydrocarboniphaga effusa* AP103	
	*Myxococcus fulvus* HW-1	*Ketobacter alkanivorans* GI5	
	*Myxococcus fulvus* DSM 16525	*Minicystis rosea* DSM 24000	
	*Myxococcus fulvus* SK5087	*Microbulbifer mangrovi* DD-13	
	*Myxococcus hansupus* mixupus	*Myxococcus fulvus* 124B02	
	*Myxococcus macrosporus* DSM 14697	*Myxococcus fulvus* DSM 16525	
	*Nannocystis exedens* ATCC 25963	*Myxococcus hansupus* mixupus	
	*Oceanococcus atlanticus* 22II-S10r2	*Nannocystis exedens* ATCC 25963	
	*Rhizobacter gummiphilus* NBRC 109400	*Nevskia soli* DSM 19509	
	*Rhizobacter gummiphilus* NS21	*Oceanococcus atlanticus* 22II-S10r2	
	*Roseateles* sp. YR242	*Rhizobacter gummiphilus* NS21	
	*Solimonas* sp. HR-BB	*Solimonas* sp. HR-BB	
	*Steroidobacter cummioxidans* 35Y	*Spongiibacter tropicus* DSM 19543	
	*Xanthomonas* sp. 35Y	*Steroidobacter cummioxidans* 35Y	
		*Xanthomonas* sp. 35Y	
		*Zhongshania* sp. ZX-21	
Bacteria	Gram-negative	Gram-negative	Gram-positive
Mechanism of cleavage	Exo	Endo	Endo
Molecular mass	∼70 kDa	∼70 kDa	∼40 kDa
Rubber degradation product	12-oxo-4,8-dimethyltrideca-4,8-diene-1-al (ODTD) a C15 oligo-isoprenoid	Mixture of C20, C25, C30 and higher oligo-isoprenoids	Mixture of C20, C25, C30 and higher oligo-isoprenoids

The rubber oxidases identified so far include the exo-type rubber oxygenase A. Nevertheless, the exo-type of reaction is accountable for its suboptimal degradation efficiency and hinders the rapid and efficient degradation of polymers. This is presumably due to the inefficient threading of the substrate chain into the enzyme (Seidel et al., 2013). The endo-type rubber oxygenase B is capable of cleaving polyisoprene to C15 oligopolyisoprene (ODTD) (Jendrossek and Birke, 2019). RoxB can endo-cleave the carbon-carbon double bond of polyisoprene to produce oligopolyisoprenes with varying degrees of polymerization (Birke et al., 2017), and this endo-type of rubber oxidase is more suitable for polyolefin degradation systems (Zhang et al., 2022). Gibson et al. (2020) screened *Nocardioides* WS12, which is capable of degrading isoprene, from soil near willow trees, and they found that the strain’s genome also contained genes related to the degradation of rubber and speculated that it might also possess the ability to degrade NR.

## 3 Rubber biodegradation by microbial consortia

### 3.1 Advantages in the constructing functional rubber degradation consortia

Researchers have shown through experimental studies and practical applications that the degradation ability of a single strain of bacteria for NR is limited, and the speed and efficiency are very restricted, while multiple microorganisms interacting with each other for a long period of time will give better results, so they have focused on bacterial consortia. The construction of complex consortia is no longer limited to the cultivation of a single microorganism (Nguyen et al., 2020; Castañeda Alejo et al., 2022; Joseph et al., 2022). Instead, a variety of microorganisms are co-cultured in a specific environment. Through the interactions between the strains, they directly or indirectly influence the micro-ecosystem maintaining a balance. The interrelationship is either mutually beneficial or competitive, and in general, multiple microorganisms work together to accelerate and enhance the rate of rubber degradation and stabilize their maximum metabolic functions.

### 3.2 Approaches for construction of artificial microbial consortia

Studies have shown that the degradation of pollutants by consortia is superior to that of purely cultured microorganisms (Table 4). The microorganisms in the functional consortia perform different metabolic functions and accomplish the degradation of pollutants while coping with the harsh living environment through collaboration among themselves (Araújo et al., 2020; Chen et al., 2021). There are two methods to construct functional consortia: one is the natural selection method, in which the soil and water contaminated by NR are mixed, and the suspension is extracted after static sedimentation, and NR samples are added to the suspension and incubated with iterative shaking until the weight of the NR samples no longer changes, and a naturally selected functional consortium is obtained in each generation of the culture (Nguyen et al., 2020). The other method is an artificial combination method, that is, the soil or water contaminated by NR is first screened for specificity, and the functional bacteria that can use NR as the only carbon source are screened for pure culture. A variety of bacteria with the ability to degrade NR are currently screened from the phylum *Actinobacteria* (Basik et al., 2021b). Later, the screened NR degrading bacteria were used for consortium construction, and the more efficient consortium for degrading NR was selected.

**TABLE 4:** Constructed microbial consortia with a rubber degradation capacity.

Composition of microial consortia	Sources	Culture time	Rubber type	Weight loss (%)	References
*Rhodococcus* sp.and obtained from the mixed culture of top five effective strains	Soil samples collected from rubber contaminated ground	4 weeks	Natural rubber glove	19	Nawong et al. (2018)
*Enterobater cloacae*, *Microbacterium laevaniformans* and *Methylobacterium rhodesianum*	Textile effluent contaminated soil	4 days	Natural rubber	5	Krishnaswamy and Ahongsangbam (2017)
*Bacillus cohnii*, *Brevundimonas naejangsanensis*	Soil samples of the rooting part of the rubber tree	20 days	Artificial rubber	50	Muralidharan and Krishnaswamy (2016)
Enriched consortium: *Delftia tsuruhatensis* (69.1%), unclassified (15.7%), *Delftia lacustris* (3.8%), *Microvirus Enterobacteria* phage PhiX174 (2.4%), *Acidovorx wohlfahrtii* (1.1%), *Bradyrhizobium liaoningense* (1.0%), *Bradyrhizobium pachyrhizi* (0.8%), *Mesorhizobium septentrionale* (0.6%), others (5.4%)	Aged tire	20 days	Tire rubber	5	Castañeda Alejo et al. (2022)
Enriched consortium: *Gordonia* (48%), *Nocardia* (20%), *Rhodococcus* (28%), others (4%)	Mixture of soil, sludge and wastewater from rubber processing factory	14 days	Natural rubber	28	Nguyen et al. (2020)
Enriched consortium: *Arthrobacter* (68%), *Mycobacterium* (14%), others (18%)	Mixture of soil, sludge and wastewater from rubber processing factory	14 days	Natural rubber	18	
Enriched consortium: *Gordonia* (26%), *Nocardia* (34%), *Rhodococcus* (32%), others (8%)	Mixture of soil, sludge and wastewater from rubber processing factory	14 days	Natural rubber	37	
Enriched consortium: *Gordonia* (38%), *Nocardia* (24%), *Rhodococcus* (22%), *Mycobacterium* (1%), others (15%)	Mixture of soil, sludge and wastewater from rubber processing factory	14 days	Deproteinized natural rubber	15	
Enriched consortium: *Mycobacterium* (90%), *Rhodococcus* (2%), others (9%)	Mixture of soil, sludge and wastewater from rubber processing factory	14 days	Deproteinized natural rubber	48	
Enriched consortium: *Gordonia* (92%), *Rhodococcus* (2%), others (7%)	Mixture of soil, sludge and wastewater from rubber processing factory	14 days	Deproteinized natural rubber	38	
Enriched consortium: *Gordonia* (95%), *Rhodococcus* (1%), others (4%)	Mixture of soil, sludge and wastewater from rubber processing factory	14 days	Deproteinized natural rubber	35	

NR degrading bacteria screened so far have been broadly classified into two groups: the first group can produce hyaline rings on a solid medium with NR as the only carbon source; the second group can form biofilms and thus grow directly attached to the surface of NR (Hapuarachchi et al., 2016). It is important to note when constructing an artificial functional consortia: only a small fraction of microorganisms in the natural environment can grow on artificial media, so the conventional plating method alone cannot completely isolate the functional microorganisms found in an environment. These difficult to isolate microorganisms may exercise a synergistic role in the degradation of pollutants, and their absence reduces the efficiency of the functional consortia in the degradation of pollutants (Lee et al., 2013). Meanwhile, Nguyen et al. (2020) found that high microbial diversity of the consortia inhibits the degradation of NR by microorganisms. Therefore, to ensure the efficiency and to maintain the stability and harmony of functional consortia, microorganisms that are abundant in the initial environment should be incorporated alongside known functional microorganisms when constructing such consortia.

Currently, there are studies on consortia analysis usually combined with metagenomics sequencing. In 1998, Handelsman et al. (1998) introduced the concept of metagenomics in this scenario. Metagenomics refers to the advancement of individual single studies of microorganisms to study the sum of all microbes. Metagenomics does not require the isolation and pure culture of microorganisms. It no longer focuses only on the role of a single strain, overcoming the limitations of isolation and culture methods. Metagenomics enables the exploration of non-culturable microorganisms, which were previously limited by technical methods, greatly expanding the scope of microbial research (Riesenfeld et al., 2004). In addition, the significant advantages of metagenomics are the ability to identify microbial communities with low abundance, to make more genetic resources available, to emphasize the exploitation and use of non-culturable microorganisms, and to uncover microbial activities and functions at the level of the complete community.

### 3.3 Construction of rubber degrading microbial consortia using omics tools

In recent years, the investigation of rubber biodegradation mechanisms and microbial ecology has been facilitated by the increased use of genomics, metagenomics, transcriptomics, proteomics, and multiomics, made possible by the rapid development of high-throughput sequencing (Illumina, PacBio, and Nanopore). In a study by Nawong et al. (2018), single bacterial strains and mixed culture of selected bacteria were subjected to degradation experiments on NR gloves in a mineral salt medium (MSM). Following a 30-day incubation period under identical conditions, the bacteria exclusively utilized the NR glove as a carbon source, resulting in the polymer’s degradation. The experimental results demonstrated that the weight loss rate of NR gloves from mixed bacterial culture was much higher than that of NR degraded by individual strains, with the highest weight loss rate of 9.36% for single bacterial degradation and 18.82% for the constructed consortia. Nguyen et al. (2020) isolated and cultured a batch of bacteria from waste from a Vietnamese rubber processing plant and used NR film as the only carbon source in MSM medium. After 14 days of incubation, the highest weight loss of NR films reached 34% among the six groups of bacteria consortia constructed, which greatly improved the degradation of NR.

**FIGURE 2:**
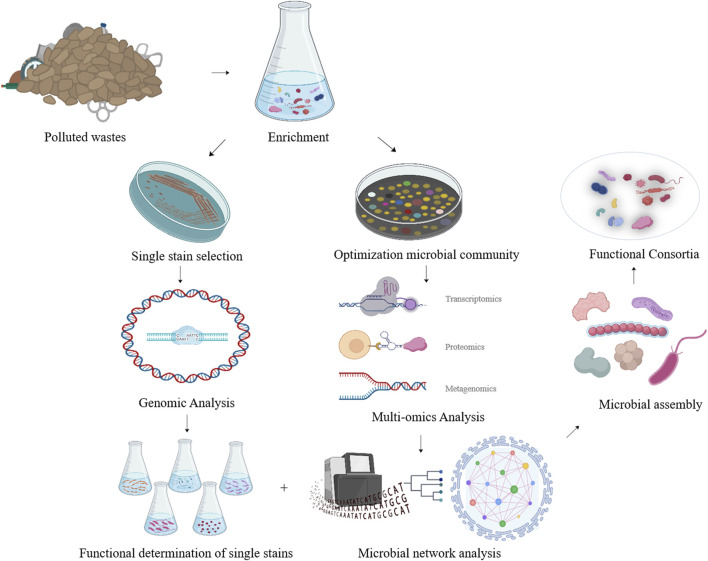
The strategy of developing efficient NR degrading microbial consortia based on multi-omics analysis of the functional microbial community achieved after enrichment.

Furthermore, by monitoring the metabolic and dynamic relationships among the functional consortia, multi-omics analysis of synthesized functional consortia could serve as a guide for subsequent manipulations of population ratios and environmental parameters (Figure 2).

## 4 Investigations and advancement of rubber biodegradation

Currently, there are several methods used to detect and analyze the ability of microorganisms to carry out the degradation of NR. The easiest and most obvious method is the weight loss measurement. To measure the weight loss of a NR sample, the entire residual rubber (including the more intact pieces and broken rubber particles) is usually separated from the rest of the culture and collected and cleaned and dried until the weight is constant (Cheng et al., 2023). The weight loss of NR is equal to the initial weight of the sample minus the residual weight. The weight of NR before degradation is recorded as w1, and the weight after washing and drying after degradation by the strain is recorded as w2: weight loss = [(w1-w2)/w1] × 100%. The advantage of the weight loss method is that it can reflect the efficiency of microbial degradation of rubber more intuitively. Theoretically, the stronger the strain’s ability to degrade NR, the higher the weight loss rate. However, the method cannot help us understand the process of microbial degradation of NR, and we cannot exclude some strains that take a long time to function. For example, some *Streptomycetes* take some time to grow and colonize on the surface of NR. Second, we can observe the surface structure of NR samples by scanning electron microscopy (SEM) to get an idea of the degradation ability of the microbial strains. The growth and degradation of the strain on NR and the structural changes on its surface before and after the degradation of NR by the strain can be observed using SEM. Residual NR samples are isolated from cultures and later washed, fixed and dried. Generally, at great magnification under SEM, the surface of the NR sample can be observed to show significant cracks, pits or holes compared to the initial state, which is considered to have some NR degradation (Handelsman et al., 1998). This method can visually observe the microscopic state of NR after degradation compared to the loss-in-weight method, which is clearer but does not help us to understand the mechanism of degradation. Furthermore, the color reaction can be employed to validate the degradation process of NR. The initial stage of microbial degradation of NR typically involves the oxidative cleavage of the double bond of cis-1,4-isoprene, resulting in the formation of aldehydes (Umsakul et al., 2012). The detection of aldehyde and ketone groups frequently serves as indications that NR samples have undergone some degree of microbial degradation. One of the easiest and fastest methods to detect the presence of aldehyde groups in the metabolites of a sample is the colorimetric reaction. This method mainly uses Schiff’s reagent. Schiff’s reagent does not show color when exposed to ketone groups but turns purple when exposed to aldehyde groups, which is a very sensitive reaction, and is often used to detect the formation of aldehyde groups. 2, 4-DNP produces a yellow precipitate when exposed to aldehyde and ketone groups, which can be used to detect the presence of aldehyde and ketone groups. Both reagents are often used for the preliminary detection of aldehyde groups during the degradation of NR samples. In addition, fourier transform infrared spectroscopy (FTIR), high-resolution mass spectrometry (HRMS) (i.e., LC-MS/LC-HRMS, GC-HRMS, MALDI-TOF), and nuclear magnetic resonance spectroscopy (NMR) also have a role in the degradation process of NR. The mode of degradation can be deduced from the functional group changes that occurred during the degradation of NR using FTIR spectroscopy. FTIR spectroscopy is often used to detect the creation of new functional groups and the disappearance of existing functional groups in a sample. By analyzing the changes of peaks and the intensity of peaks at specific locations in the FTIR spectra before and after the degradation of NR samples, we can know the changes of functional groups during the degradation of the samples. These data help us to verify that microorganisms have the ability to degrade NR samples and to speculate on the reactions that may occur during the degradation process. In general, the first functional groups that studies focus on are carbonyl, ester, ethylene and carbon-carbon double bonds. It has been shown that these groups usually change whenever microbial activity on the substrate surface is detected (Sudhakar et al., 2008; Nayanashree and Thippeswamy, 2015a). Additionally, studies focus on the changes of aldehyde and ketone groups. During the degradation of NR, there is a release of aldehydes and ketones. The presence of these aldehyde and ketone groups confirms the degradation of NR by the strain due to degradation. The combined observation and analysis of the above groups can collectively indicate whether the strains have a NR degradation capacity. By identifying the location and quantity of the various forms of C and H atoms within organic matter, NMR can be utilized to determine the structure of metabolites generated during the degradation. The metabolites produced during the degradation of NR can be inferred from the information from the NMR hydrogen and carbon spectra of the samples (Handelsman et al., 1998), and if the results show the presence of aldehyde and ketone groups, the degradation of NR can thus be determined.

## 5 Prospects for rubber degradation in the future

### 5.1 Current bottlenecks and future research directions in rubber biodegradation

In the majority of recent investigations, it has been observed that the biodegradation process of NR has shown a rather slow rate, despite the successful identification and isolation of a limited number of functional bacteria capable of degrading this material (Bosco et al., 2018; Joseph et al., 2022; Sarkar et al., 2022; Cheng et al., 2023). The duration of microbial culture has shown a range of 30–120 days, but assessment methods such as weight loss or SEM have only identified little deterioration. The main limitation of existing research is the absence of consistent standards and dependable techniques for accurately quantifying the efficacy of deterioration. For this reason, establishing standardized procedures for quantifying the degradation of NR is essential for future research evaluating the biodegradability of microorganisms.

In addition, many chemical additives, including vulcanization accelerators, antioxidants, retarders, and their breakdown products, are utilized in industrial rubber products, including tires. These additives are essential in commonly used rubber materials because they prolong the material’s life and durability by avoiding breakdown (Altenhoff et al., 2019; Kawakami et al., 2022). Antioxidants were shown to be the most interfering with *in vitro* depolymerization, and the substance’s hydrophobic nature, as well as the huge amounts of additives present, prevent considerable biodegradation rates. For this reason, it is crucial to identify specific critical additives found in common rubber in order to enhance the microbial or enzymatic degradation process, for instance by facilitating or establishing the desired pretreatment of rubber materials. Conversely, the incorporation of additive-degrading microorganisms into a functional consortia could enhance degradation capability through synergy with other bacteria in the consortia. As a result of their low enzyme production efficiency and straightforward enzyme systems, single stains are incapable of effectively combating the complex rubber degradation process. It is recommended that future research endeavors concentrate on both the discovery of novel, efficient enzymes/genes for rubber degradation and the investigation of degradation mechanisms. Furthermore, the integration of genetic engineering and enzyme engineering methodologies may facilitate the development of microorganisms that produce rubber-degrading enzymes with enhanced activities. Temperature, ultraviolet light, dissolved oxygen, humidity, pH, and a variety of biotic and abiotic factors (microbial abundance and diversity, microbial enzymatic activity, etc.) influence the microbial rubber degradation process. In order to determine the optimal conditions for rubber biodegradation, it is necessary to take into account a variety of factors, such as environmental parameters and condition of the culture. Despite our increased knowledge of enzyme activity, we know little about rubber substrate enzyme action and the microbial, molecular, and environmental variables that affect it. In order to fully understand the microbial diversity, biofilm-formation mechanisms, and biodegradation capacity of rubber waste, multi-omics studies should be conducted.

### 5.2 Possible strategies for rubber-valorization

Despite the severe environmental damage caused by conventional rubber disposal methods, it is intriguing that new rubber valorization technologies that are economically and ecologically viable are being proposed and developed to meet the growing amount of rubber waste. Rubber waste is valorized through energy recovery, recycling, upcycling, or biodegradation in order to produce new, viable products or dispose of it in an environmentally friendly manner. Incineration and landfilling are currently the prevailing and efficacious approaches for managing rubber and its byproducts. For instance, a portion of the heat produced during the incineration procedure can be recovered and harnessed to generate electricity or heat. Unfortunately, traditional recycling or direct incineration has many disadvantages, namely, the tedious process of collection, sorting and cleaning, as well as limited recycling time, excessive costs, emission of harmful residues, and unsuitability for use in other products. Instead, chemical or biological methods of converting rubber waste, which are polymeric molecules, into smaller molecules and transforming these molecules into new and valuable products are emerging as an attractive and more environmentally friendly route to valorization (Stevenson et al., 2008; Andler, 2020; Bosco and Mollea, 2021). By utilizing metagenomics sequencing, one can functionally annotate related genes and assess the diversity and relative abundance of microorganisms in a given community. It was disclosed that as the microbial diversity of artificially constructed consortia decreases, the number of strains capable of degrading NR increases, thereby enhancing the community’s efficiency (Castañeda Alejo et al., 2022).

**FIGURE 3:**
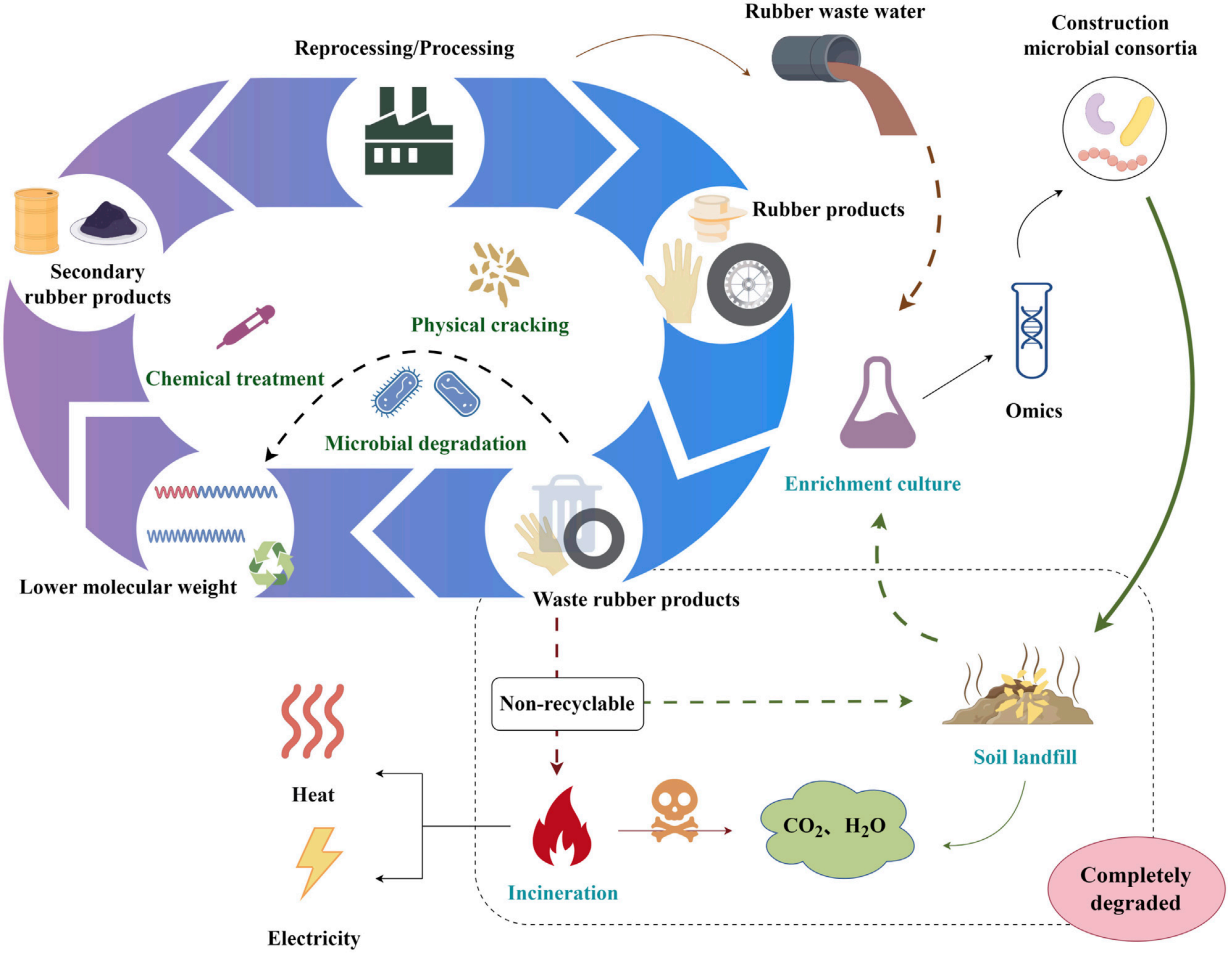
A schematic overview of the bio-upcycling of rubber waste. The figure was generated by Figdraw (www.figdraw.com).

Integrating microbial consortia with multi-omics might inspire a novel rubber waste bio-upcycling technique (Figure 3). Multi-omics will illuminate rubber biodegradation metabolic pathways and microbial community interactions. The synthesis and logical design of the composition and metabolic pathways of microbial consortiums would facilitate biotransformation into a greater variety of rubber biodegradation products, thereby expanding the product line of rubber waste upcycling.

## 6 Outlook and perspective

The gradual and widespread use of NR has resulted in a growing problem of rubber waste pollution and pollutants worldwide, which is undoubtedly a huge challenge for environmental management. Traditional rubber disposal methods are not effective in solving this problem, which is becoming more serious. On the biodegradation of NR, rather, recent research has produced fresh insights. An expanding number of microorganisms implicated in the degradation process must be identified, as well as novel approaches to the sustainable dispersal of rubber wastes. NR is the most widely used rubber, and the study of its biodegradation has received a great deal of attention in the past few years. The investigation of NR degradation by microorganisms, including bacteria and fungi, has been the focus of considerable research. Despite this, progress in the study of microbial degradation of NR remains limited, particularly with regard to the identification of specific NR depolymerizing enzymes and the elucidation of NR degradation mechanisms. Recent studies have examined the limitations encountered by individual strains in their attempts to degrade NR, and progressive efforts have been made to combine several strains to develop microbial consortia. Advancements have been achieved in the developing functional consortia or synthesized co-cultures for greater efficacy. The advantages of functional microbial consortia in degrading NR and its waste surpass those of single strains. Nevertheless, the development of artificial functional consortia, which is the subject of this review, is impeded by the lack of comprehension regarding the by-products and mechanisms of NR degradation.

Currently, researchers have isolated and screened NR degrading strains from various sources and used the weight loss method to detect weight change, Schiff’s reagent to detect whether aldehyde groups are produced during the degradation of NR, SEM to observe the surface structure of NR before and after degradation, and Fourier infrared spectroscopy and nuclear magnetic resonance instruments to investigate the functional groups and motifs of NR. None of the relevant testing methods confirmed the degradation of NR by microorganisms. However, the degradation of NR by a single microorganism takes more time and has an inferior degradation rate and capacity compared to that of a complex consortia. With the advancement and development of metagenomics sequencing, we can consider combining the two. Metagenomics sequencing helps to construct the microbial composition in the consortia and discover and identify the microbial consortia with lower abundance to make more genetic resources available. It emphasizes the development and utilization of non-culturable microorganisms and discovers the functions of microorganisms from the complete community level. Therefore, it is more beneficial to explore the role of microorganisms in the degradation of NR based on metagenomics sequencing results to construct an efficient NR degradation microbial consortium.

The integration of omics/meta-omics techniques, including genomic, transcriptomic, metagenomic, and others, can facilitate the disclosure of microbial diversity. The identification of the microbial communities and critical pathways/genes involved in the degradation of NR is made possible through omics analyses, which also serve as a foundation for the development of functional consortiums. In conclusion, we deliberated on the current challenges and obstacles encountered in the field of NR biodegradation research, as well as provided some perspectives on potential areas for future investigation. At present, the development of efficient microbial consortia encounters numerous challenges stemming from the inadequate investigation of the biodegradation mechanisms underlying NR. Furthermore, future research may encounter difficulties concerning the regulation of coexistence among microorganisms and the safety and stability of synthesized microbial consortiums. The dynamic characteristics of microbial communities, biological variability, and a multitude of other uncertainties arising from microbial communities constitute obstacles for future research.

## 7 Conclusion

Although attention to microbial degradation of NR has continued for many years, single microorganisms that have been discovered are difficult to take on the task of treating rubber wastes. The emergence of microbial consortia has solved the survival problem of individual strains to some extent and further improved the degradation efficiency. How to construct a stable and efficient consortium is the key to solve the microbial degradation of NR. Combining the method of screening single functional strains with omics tools can not only discover more functional genes involved in the degradation of NR and clarify the metabolic pathway of microbial degradation of NR; it can also help us understand the structural composition of microorganisms in the sample. The construction of microbial consortia also faces many difficulties, such as how to rationally utilize the complex interspecies relationship between microorganisms, overcome the antagonistic effect of local microorganisms on foreign microorganisms, and achieve stable and efficient degradation goals, all of which need to be solved urgently.
